# Supplementary material for: An exploration of proactive health oriented symptom patterns in patients undergoing percutaneous coronary intervention with stent implantation: A mixed-methods study protocol
Source: PLoS One. 2023 Oct 5;18(10):e0292285. doi: 10.1371/journal.pone.0292285 (PMC10553226; doi:10.1371/journal.pone.0292285)
Supplement: S1 Checklist — (DOCX) [file pone.0292285.s001.docx]

Good Reporting of A Mixed Methods Study (GRAMMS) checklist

Title: Improving quality of care for pregnancy, perinatal and newborn care at district and sub-district public health facilities in three districts of Haryana, India: An implementation study

| Guideline | Section: page |
| --- | --- |
| Describe the justification for using a mixed methods approach to the research question | Methods- under Study design overview, page 8-9 |
| Describe the design in terms of the purpose, priority and sequence of methods | Methods- under Study design overview, page 8-9 |
| Describe each method in terms of sampling, data collection and analysis | Methods- under Sample and study setting, Page 10 and 14  Methods- under Data collection, Page 11-13 and 14-15  Methods- under Analysis of data, Page 13 and 15 |
| Describe where integration has occurred, how it has occurred and who has participated in it | Methods- under Integration of the data, Page 16-17 |
| Describe any limitation of one method associated with the present of the other method | Discussion, Page 18-19 |
| Describe any insights gained from mixing or integrating methods | Discussion, Page 17-18 |

*Ref: O'Cathain A, Murphy E, Nicholl J. The quality of mixed methods studies in health services research. J Health Serv Res Policy. 2008;13: 92-98*
